# Triplet systemic therapy for hormone-sensitive prostate cancer: a critical review with a multidisciplinary approach

**DOI:** 10.3389/or.2025.1599292

**Published:** 2025-07-25

**Authors:** Almudena Zapatero, Teresa Alonso-Gordoa, Alfredo Rodríguez Antolín, Felipe Couñago, Noelia Sanmamed, Mario Domínguez Esteban, Marta López Valcárcel, Ray Manneh, Ángel Borque-Fernando, Nuria Sala González, Pablo Maroto

**Affiliations:** ^1^ Radiation Oncology Department, Health Research Institute, Hospital Universitario de La Princesa, Madrid, Spain; ^2^ Medical Oncology Department, Ramón y Cajal University Hospital, Madrid, Spain; ^3^ Urology Service, Hospital Universitario 12 de Octubre, Madrid, Spain; ^4^ GenesisCare Radiation Oncology Department, San Francisco de Asís University Hospital and Vithas La Milagrosa University Hospital, Madrid, Spain; ^5^ Department of Medicine, Faculty of Medicine, Health and Sport Sciences, Universidad Europea de Madrid, Madrid, Spain; ^6^ Radiation Oncology Department, Clínico San Carlos Hospital, Madrid, Spain; ^7^ Urology Service, Marqués de Valdecilla University Hospital, Santander, Spain; ^8^ Radiation Oncology Department, Puerta de Hierro Majadahonda University Hospital, Madrid, Spain; ^9^ Society of Oncology and Hematology of Cesar, Valledupar, Colombia; ^10^ Urology Service, Miguel Servet University Hospital, IIS-Aragón, Zaragoza, Spain; ^11^ Medical Oncology Service, Catalan Institute of Oncology, Josep Trueta Hospital, Girona, Spain; ^12^ Medical Oncology Service, Hospital de la Santa Creu i Sant Pau, Barcelona, Spain

**Keywords:** androgen deprivation therapy, androgen receptor-targeted therapy, docetaxel, hormone-sensitive prostate cancer, metastatic prostate cancer

## Abstract

This article aims to critically evaluate the evidence for triplet therapy consisting of androgen deprivation therapy (ADT), docetaxel and a second-generation androgen receptor pathway inhibitor ([ARPI]; abiraterone, enzalutamide, darolutamide or apalutamide) in patients with metastatic hormone-sensitive prostate cancer (mHSPC), and what this evidence reveals regarding the use of these treatments in clinical practice. A search of PubMed, Medline, Embase, Cochrane, Scopus and Web of Science was conducted in April 2024 to identify relevant prospective and retrospective observational trials, randomized controlled trials (RCTs) and meta-analyses. The search identified 52 relevant articles: six full articles and 31 abstracts based on three RCTs, one observational study and 14 meta-analyses. Abiraterone- or darolutamide-containing triplet therapy was significantly better than ADT + docetaxel for improving overall survival in all study populations, particularly subgroups with high-volume and/or synchronous disease. The tolerability of ADT + docetaxel and triplet therapy were similar with most adverse events related to docetaxel. There were no data comparing triplet therapy with ADT + ARPI doublet therapy. Triplet therapy appears to be the most effective first-line regimen for men with mHSPC, good performance status and high-volume and synchronous metastases. Darolutamide-based triplet therapy may also be of benefit in other patients with high- or low-risk disease. Careful consideration of the risks and benefits are required to determine which patients can be spared from receiving docetaxel and rather be treated with alternative regimens.

## 1 Introduction

It is estimated that 1.5 million men were diagnosed with prostate cancer in 2022 (1), and the global incidence is estimated to almost double in the next 5 years with changing demographic trends (2). Prostate cancer is the fourth most common cancer and the eighth leading cause of cancer deaths (3). Approximately 10%–15% of men with prostate cancer have metastatic hormone-sensitive prostate cancer (mHSPC) (4).

Over the past 10–15 years, the systemic treatment of mHSPC has been evolving from treatment with androgen deprivation therapy (ADT) alone, to doublet therapy consisting of ADT + docetaxel or ADT + a second-generation androgen receptor pathway inhibitor (ARPI), and more recently to triplet therapy with ADT + second-generation ARPI + docetaxel (5). Both European and American guidelines now include triplet therapy as first-line recommendations for selected patients with mHSPC (6-8).

The aim of the current article is to critically evaluate the evidence for triplet therapy in mHSPC, and what this evidence tells us about how to use triplet therapy in clinical practice, focusing on identifying the optimal patient characteristics for this treatment alternative.

## 2 Materials and methods

We conducted a literature search of PubMed, Medline, Embase, Cochrane, Scopus and Web of Science on 17 April 2024 to identify potentially relevant studies. The search strategy used a range of MeSH terms designed to identify studies in which ADT, ARPIs and docetaxel were all used in patients with mHSPC (see the Supplementary Material for full details of search strategies). All clinical trial types were considered for inclusion (observational and randomized controlled trials [RCTs]; prospective and retrospective), but only English-language articles were included. Additional articles were identified based on the authors’ experience in the area and a number of articles were added at the suggestion of the peer reviewers. Reviews, editorials, news items, case reports and correspondence were excluded, but bibliographies were reviewed for potentially relevant data. While priority was given to articles published in peer-reviewed journals, conference abstracts were considered for inclusion if they presented information that may help to clarify treatment decisions. No date limits were set, but only conference abstracts published since January 2021 were reviewed for potential inclusion.

## 3 Results

**FIGURE 1:**
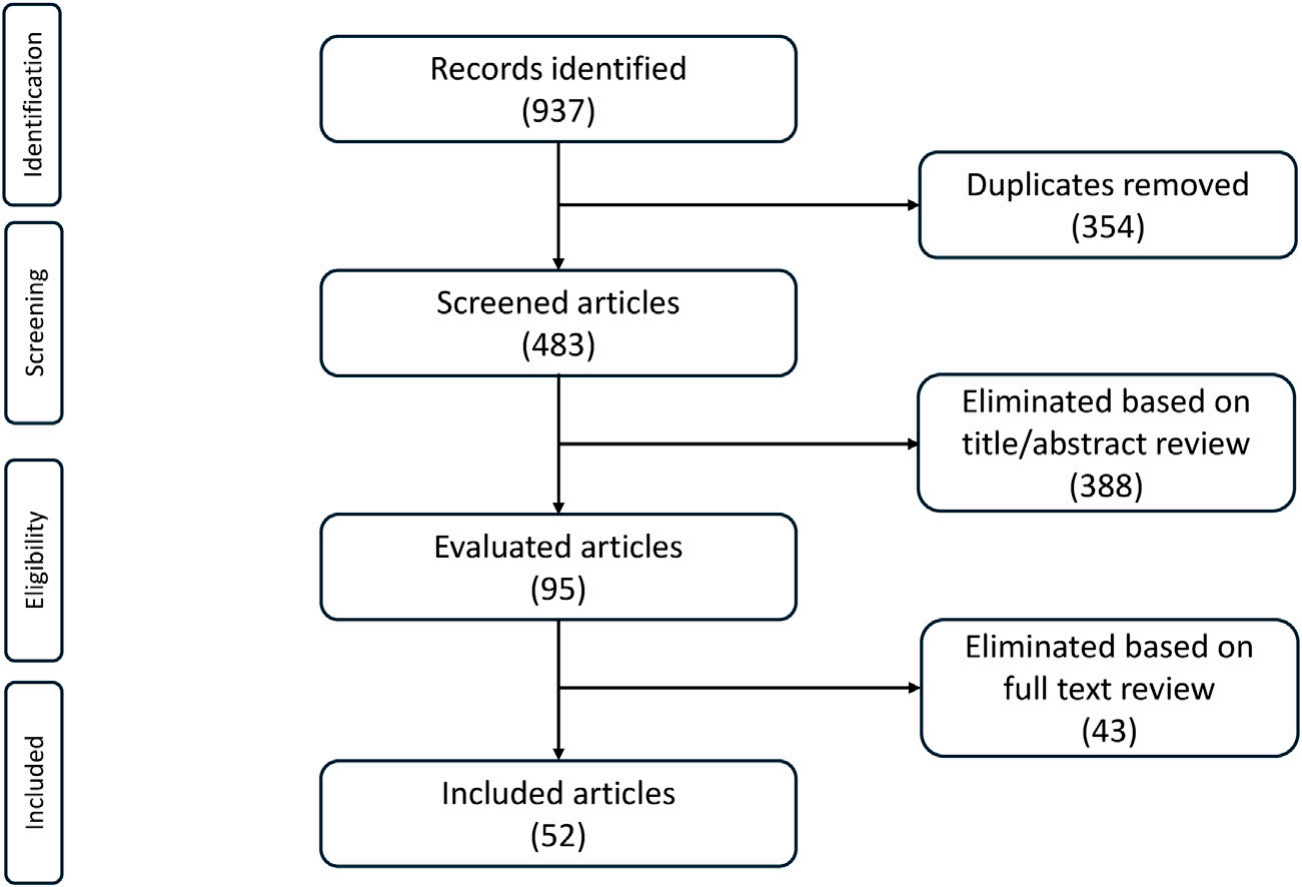
Article identification and inclusion.

Our search identified 483 articles, which were investigated for inclusion. Of these, 95 were considered potentially relevant and 52 were considered relevant (Figure 1). Three major RCTs (ARASENS, ENZAMET and PEACE-1) collectively generated seven clinical research articles (9-14), 31 abstracts (15-45) and one congress highlight with abstract (46). We also identified one observational study (47) and 14 meta-analyses (48-61).

### 3.1 Randomized controlled trials

#### 3.1.1 Design

Of the three RCTs that comprised most of the articles in our search, only the ARASENS study set out to compare triplet therapy and doublet therapy from the start (12). The PEACE-1 and ENZAMET studies began using ADT alone (as the standard of care [SOC]) for background therapy but later included ADT + docetaxel as an option once this combination became the SOC (11, 13, 14).

Patient inclusion/eligibility criteria were similar but the studies had different designs (Table 1). ARASENS had a straightforward randomized, double-blind, placebo-controlled design, with two treatment arms; patients in both the active treatment group and control group received ADT + docetaxel (12), but one received darolutamide and the other placebo, i.e., this was a comparison of doublet therapy with ADT + docetaxel *versus* triplet therapy.

**TABLE 1:** Study designs of the PEACE-1, ARASENS and ENZAMET studies.

	Peace-1 (13)	Arasens (12)	Enzamet (11, 14)
Phase	3	3	3
Design	Multicenter (77), randomized (1:1:1:1), open-label, active-controlled with 2 × 2 factorial design	Multicenter (286), randomized (1:1), double-blind, placebo-controlled	Multicenter (83), randomized (1:1), open-label, active-controlled
Patient inclusion	Age ≥18 years; histologically or cytologically confirmed prostate adenocarcinoma with metastases detected by imaging; ECOG PS 0–1 or 2 due to bone pain; had received ADT for ≤3 months before randomization and had ≥6 weeks between initiation of ADT and first docetaxel dose	Age ≥18 years; histologically or cytologically confirmed prostate cancer with metastases detected by imaging; ECOG PS 0 or 1; candidates for ADT and docetaxel according to investigator judgement	Age ≥18 years; histologically or cytologically confirmed prostate adenocarcinoma with metastases detected by imaging; ECOG PS 0–2
Key patient exclusion criteria	Prior cytotoxic chemotherapy or biological therapy for prostate cancer; uncontrolled hypertension; concomitant use of strong CYP3A4 inhibitors	Only regional lymph node involvement; receipt of ADT >12 weeks before randomization; receipt of second-generation ARPI, chemotherapy or immunotherapy for prostate cancer before randomization; RT within 2 weeks before randomization	History of seizure or any condition that may predispose to seizure
Treatment groups	ADT ± docetaxel as SOC vs. SOC + RT vs. SOC + abiraterone^a^ 1,000 mg OD vs. SOC + RT + abiraterone^a^ 1,000 mg once daily	ADT + docetaxel (+ steroids as needed) + darolutamide 600 mg BID vs. matching placebo	ADT ± docetaxel + enzalutamide vs. ADT ± docetaxel + nonsteroidal ARPI^b^
Stratification factors	Study site, ECOG PS (0 vs. 1–2), type of ADT (GnRH antagonist vs. GnRH agonist vs. orchiectomy), planned administration of docetaxel (yes vs. no), metastatic status (only lymph nodes vs. bone vs. visceral)	Metastatic stage (M1a, M1b or M1c), ALP level (below, at or above normal range)	Study site, disease volume (high vs. low^c^), planned use of docetaxel (yes vs. no), planned use of bone antiresorptive therapy (yes vs. no), Adult Comorbidity Evaluation-27 score (0–1 vs. 2–3)
Primary endpoint(s)	OS and radiographic PFS (co-primary endpoints)	OS	OS
Secondary endpoints	CRPC-free survival, serious genitourinary event-free survival, prostate cancer-specific survival, time to next skeletal-related event, PSA response rate, prognostic study of serum PSA 6–8 months after initiation of therapy, time to pain progression, time to chemotherapy for CRPC, QoL, change in BMD, correlation of biomarkers with outcomes, event rate per 100 PY of treatment, toxicity	Time to CRPC, time to pain progression, symptomatic skeletal event-free survival, time to first symptomatic skeletal event, time to initiation of subsequent systemic antineoplastic therapy, time to worsening disease-related physical symptoms, time to initiation of opioid treatment for ≥7 days, safety	PSA PFS, clinical PFS, health-related QoL, health outcomes relative to costs, safety
PSA assessment frequency	Every 6 months	Every 12 weeks	Every 3 months
Number of patients	1,172 (SOC: n = 296, SOC + RT: n = 293, SOC + abiraterone: n = 292, SOC + abiraterone + RT: n = 291)	1,305 (darolutamide: n = 651, placebo: n = 654)	1,125 (enzalutamide: n = 563, control: n = 562)
Median duration of follow-up	4.4 years (for OS) and 3.5 years (for radiographic PFS)	43.7 vs. 42.4 months	68 months

^a^ Abiraterone was administered with prednisone 5 mg BID.

^b^ Bicalutamide, nilutamide or flutamide.

^c^ High-volume disease defined as ≥4 bone lesions with ≥1 beyond the vertebrae and pelvis or visceral metastases or both; low-volume disease was defined as the absence of high-volume disease.

ADT, androgen deprivation therapy; alkaline phosphatase; ARPI, androgen receptor pathway inhibitor; BID, twice daily; BMD, bone mineral density; CRPC, castration-resistant prostate cancer; Eastern Cooperative Oncology Group performance status; GnRH, gonadotropin hormone-releasing hormone; PSA, prostate-specific antigen; OD, once daily; OS, overall survival; PFS, progression-free survival; PY, patient-years; QoL, quality of life; RT, radiotherapy; SOC, standard of care.

PEACE-1 was open-label and included four treatment groups using a 2 × 2 factorial design: 1) SOC alone; 2) SOC + radiotherapy; 3) SOC + abiraterone (and prednisone); and 4) SOC + abiraterone + radiotherapy (13). At the start of the study, SOC was ADT, but a protocol modification allowed physicians to start using ADT + docetaxel as SOC. Therefore, not all patients received docetaxel as SOC; of the 583 patients assigned to abiraterone (with or without radiotherapy), 355 (60.9%) received the triplet regimen of ADT + docetaxel + abiraterone.

ENZAMET was also open-label, with patients randomized to SOC + enzalutamide or SOC + a first-generation nonsteroidal ARPI (bicalutamide, flutamide or nilutamide) (11, 14). After enrolment of the first 88 patients, investigators were given the option to add open-label docetaxel to ADT as SOC. As a result, some of the patients (n = 483; 43%) received triplet therapy, of whom 243 received enzalutamide and 240 received a nonsteroidal ARPI. The rest of the patients in this study (n = 642) received doublet therapy with either enzalutamide + SOC or a nonsteroidal ARPI + SOC. Therefore, while patients in the ENZAMET study received doublet or triplet therapy, the study was not designed to compare outcomes in these two groups; rather, it was designed to compare outcomes between patients receiving enzalutamide and those receiving a first-generation ARPI. Moreover, patients receiving doublet therapy in this study received ADT with either enzalutamide or nonsteroidal ARPI; none received ADT + docetaxel.

All three studies had overall survival (OS) as a primary endpoint (11-13), but the PEACE-1 study had a co-primary endpoint of radiographic progression-free survival (PFS) (13).

Regarding patient characteristics, the population of the ARASENS trial had high-risk disease (78.2% Gleason score ≥8, 86.1% synchronous disease and 77.0% high-volume disease) (12, 40). All patients included in the PEACE-1 trial had synchronous disease and 57%–65% also had high-burden disease, so therefore were also at high risk overall (13). In ENZAMET, 60.6% of patients had synchronous disease, 53.5% had high-volume disease and 57%–60% had a Gleason score ≥8; 61% of patients with high-volume disease received docetaxel *versus* 27% of low-volume disease patients (11).

#### 3.1.2 Results–survival

In all three studies, the treatment groups that included a next-generation ARPI had significantly better OS than the groups without this therapy (11-14).

In ARASENS, the only study specifically designed to compare doublet and triplet therapy as a predefined objective, triplet therapy containing darolutamide reduced the risk of death by 32.5% compared with ADT + docetaxel (hazard ratio [HR] 0.68, 95% confidence interval [CI] 0.57–0.80; p < 0.001; Table 2), even though 76% of patients in the doublet therapy group received subsequent systemic therapies (12). Triplet therapy was also associated with a significantly reduced risk of most secondary endpoints including time to castration-resistant prostate cancer (CRPC; HR 0.36, 95% CI 0.30–0.42; p < 0.001; Table 2), time to pain progression (HR 0.79, 95% CI 0.66–0.95; p = 0.01; Table 2), symptomatic skeletal event-free survival (HR 0.61, 95% CI 0.52–0.72; p < 0.001), time to first symptomatic skeletal event (HR 0.71, 95% CI 0.54–0.94; p = 0.02), and time to initiation of subsequent antineoplastic therapy (HR 0.39, 95% CI 0.33–0.46; p < 0.001). There was no significant difference between triplet and ADT + docetaxel doublet therapy in the time to worsening of disease-related symptoms (12). Median time to initiation of opioid use for ≥7 days was not reached in either group, but there was a trend towards earlier initiation of opioids in the triplet *versus* the doublet therapy group (HR 0.69, 95% CI 0.52–0.91).

**TABLE 2:** Primary and selected secondary endpoint results of the PEACE-1, ARASENS and ENZAMET studies.

	Median (months)		HR	P-value
	Control	Triplet therapy		
ARASENS (12)	ADT + docetaxel (n = 654)	ADT + docetaxel + darolutamide (n = 651)		
OS	48.9	NR	0.68 (95% CI 0.57–0.80)	<0.001
Time to CRPC	19.1	NR	0.36 (95% CI 0.30–0.42)	<0.001
Time to pain progression	27.5	NR	0.79 (95% CI 0.66–0.95)	0.01
PEACE-1 (13)	ADT + docetaxel ± RT (n = 355)	ADT + docetaxel + abiraterone^a^ ± RT (n = 355)		
OS	52.8	NR	0.75 (95.1% CI 0.59–0.95)	0.017
Radiographic PFS	24.0	54.0	0.50 (99.9% CI 0.34–0.71)	<0.0001
CRPC-free survival	16.8	38.4	0.38 (95% CI 0.31–0.47)	<0.0001
Prostate cancer-specific survival	56.4	NR	0.69 (95% CI 0.53–0.90)	0.0062
ENZAMET (11, 14)	ADT + docetaxel + first-generation ARPI (n = 250)	ADT + docetaxel + enzalutamide (n = 253)		
OS	62.0	NR	0.73 (95% CI 0.55–0.99)	NA
PSA PFS	68.0	22.0	0.44 (95% CI 0.38–0.52)	NA
Clinical PFS	81.0	25.0	0.45 (95% CI 0.39–0.53)	NA
Prostate cancer-specific survival	NA	NA	0.67 (95% CI 0.54–0.82)	NA

^a^ Abiraterone was administered with prednisone 5 mg BID.

ADT, androgen deprivation therapy; BID, twice daily; CI, confidence interval; CRPC, castration-resistant prostate cancer; HR, hazard ratio; OS, overall survival; PFS, progression-free survival; RT, radiotherapy.

**FIGURE 2:**
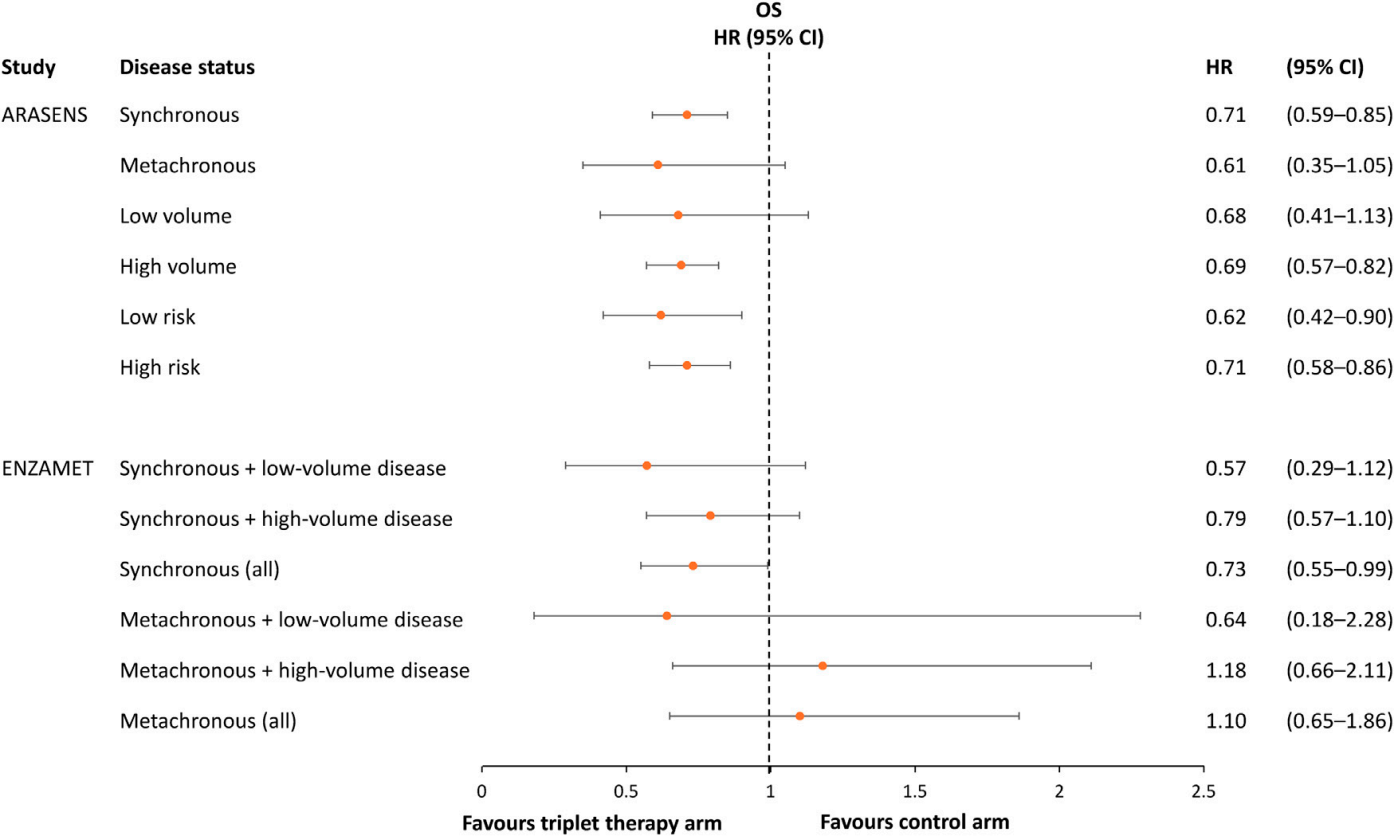
Hazard ratio and 95% confidence intervals for overall survival in subgroups of patients in the ARASENS and ENZAMET studies, stratified by metastatic status/volume and/or disease risk (9, 12, 14).

ENZAMET, which compared enzalutamide with first-generation ARPIs in combination with ADT ± docetaxel, found that the enzalutamide-containing regimen reduced the risk of death by 30% (HR 0.70, 95% CI 0.58–0.84; p < 0.0001) in the overall study cohort at final analysis (14). Enzalutamide-containing treatment was also associated with a significantly reduced risk of most secondary endpoints including time to prostate-specific antigen (PSA) progression (HR 0.44, 95% CI 0.38–0.52), time to clinical progression (HR 0.45, 95% CI 0.39–0.53), and prostate cancer-specific survival (HR 0.67, 95% CI 0.54–0.82) (14). However, in a pre-specified subgroup analysis of patients with early planned use of docetaxel (the triplet therapy group), the difference in OS between the enzalutamide group and control group did not reach statistical significance (HR 0.82, 95% CI 0.63–1.06). On the one hand, the use of docetaxel was at the investigator’s discretion, and patients who were selected for triplet therapy in this study had worse prognostic features than those who received doublet therapy, specifically a higher proportion of patients receiving triplet therapy had synchronous and/or high-volume disease. On the other hand, in the high-risk subgroup (patients with synchronous high-volume disease), older patients and those with comorbidities were less likely to receive docetaxel (14). A pre-specified subgroup analysis showed that triplet therapy containing enzalutamide significantly improved OS in patients with synchronous metastases compared with triplet therapy containing a first-generation ARPI, but not in those with metachronous disease (Figure 2) (14).

The subgroup findings from ENZAMET are consistent with results in the ARASENS study, in which the effect of triplet *versus* ADT + docetaxel doublet therapy on OS remained significant in the group with synchronous metastases but not those with metachronous metastases (9). In ARASENS, triplet therapy significantly improved OS in patients with high-volume disease (using the definition from the CHAARTED study, i.e., visceral metastases and/or ≥4 bone metastases with ≥1 beyond the vertebral column and pelvis (62)) and in patients with low- or high-risk disease (high-risk defined as two of the following criteria: Gleason score ≥8, ≥3 bone metastases and presence of measurable visceral metastasis (63)), but not those with low-volume disease (Figure 2) (9).

In the PEACE-1 study, 60.5% of patients received docetaxel as SOC, so the results in this subgroup allowed a comparison of triplet therapy with ADT + abiraterone + docetaxel (±radiotherapy; n = 355) and doublet therapy with ADT + docetaxel (±radiotherapy; n = 355) (13). Triplet therapy significantly improved the two co-primary endpoints of OS and radiographic PFS compared with ADT + docetaxel. The adjusted HR for OS was 0.75 (95.1% CI 0.59–0.95; p = 0.017) and the adjusted HR for radiographic PFS was 0.50 (99.9% CI 0.34–0.71; p < 0.0001).

Secondary endpoints of CRPC-free survival and prostate cancer-specific survival were also significantly better in the triplet than doublet therapy groups, with median CRPC-free survival of 3.21 *versus* 1.45 years in the triplet *versus* ADT + docetaxel doublet therapy groups, respectively (HR 0.38, 95% CI 0.31–0.47; p < 0.0001). Median prostate cancer-specific survival was not reached in the triplet therapy group but was 4.72 years in the doublet therapy group (HR 0.69, 95% CI 0.53–0.90; p = 0.0062) (13).

**FIGURE 3:**
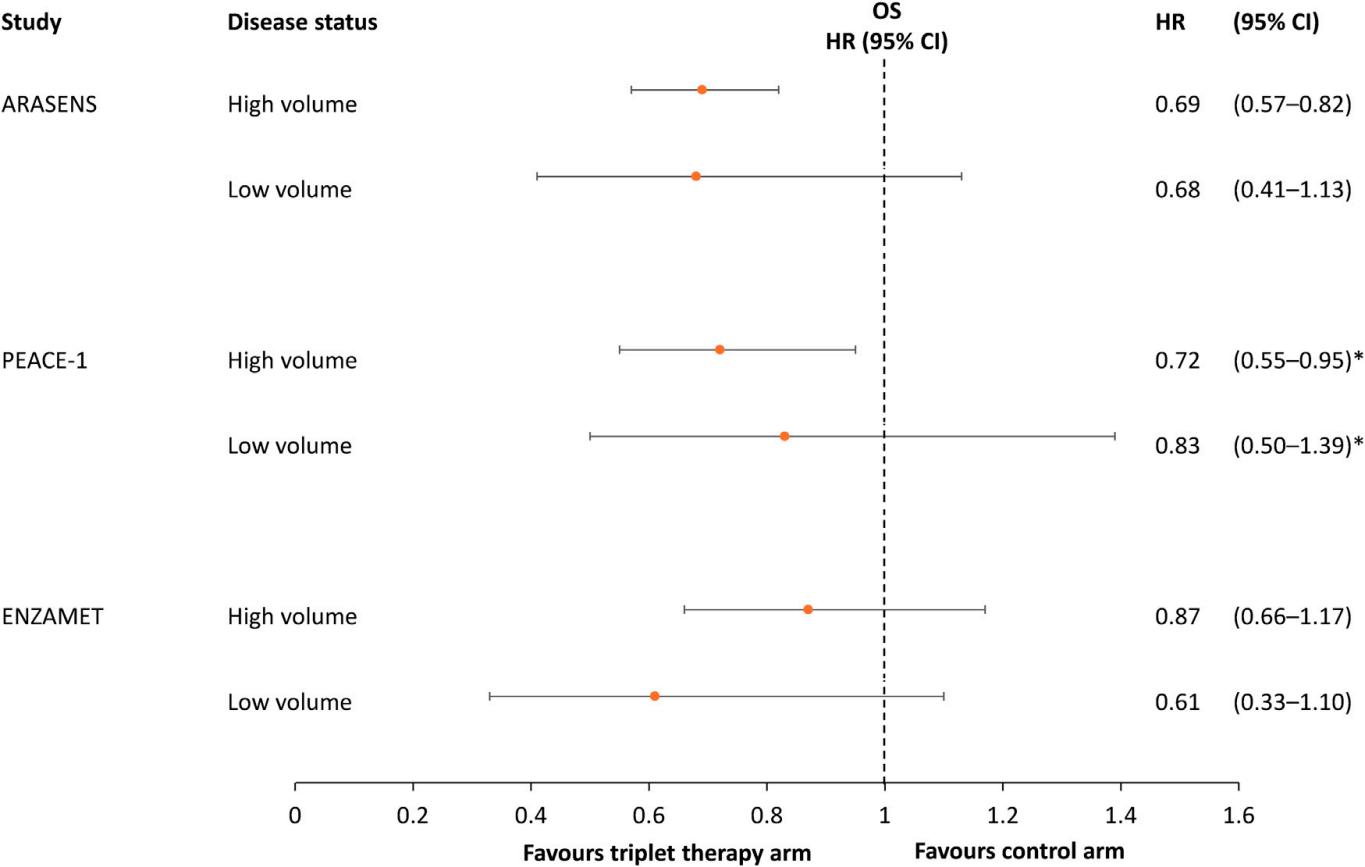
Hazard ratio and 95% confidence intervals for overall survival in the ARASENS, PEACE-1 and ENZAMET studies, stratified by metastatic volume (12, 13, 14). *95.1% CI.

Consistent with the results of ARASENS, triplet therapy in PEACE-1 was associated with a significant improvement in OS in the subgroup of patients with high-volume metastatic disease but not in those with low-volume disease (Figure 3) (13). However, the other co-primary endpoint of radiographic PFS showed a significant improvement with triplet *versus* ADT + docetaxel doublet therapy in patients with low- or high-volume metastatic disease, with an HR of 0.58 (99.9% CI 0.29–1.15; p = 0.0061) in the low-volume group and 0.47 (99.9% CI 0.30–0.72; p < 0.0001) in the high-volume group (13).

Overall, triplet therapy regimens demonstrated superior survival benefits in the key trials *versus* doublet therapy in terms of radiographic PFS, time to CRPC, pain progression and remaining free from skeletal events, and the need for subsequent antineoplastic therapy.

#### 3.1.3 Results–demographic subgroups

The preliminary results of a PEACE-1 subanalysis suggested that the magnitude of the benefit of adding abiraterone to SOC decreased with age (44). However, the magnitude of the improvement in radiographic PFS with triplet *versus* ADT + docetaxel doublet therapy was similar in men aged ≥70 years (HR 0.55, 95% CI 0.29–1.04) and those aged <70 years (HR 0.5, 95% CI 0.33–0.78), whereas younger men tended to derive a greater OS benefit (HR 0.71, 95% CI 0.52–0.95) than men aged ≥70 years (HR 0.80, 95% CI 0.53–1.2) (44). This was not the case in the ENZAMET trial, in which patients aged <70 and ≥70 years both derived a similar benefit from triplet therapy (43). The magnitude of the OS benefit associated with enzalutamide-containing triplet therapy relative to a nonsteroidal antiandrogen-containing triplet therapy in these age subgroups did not reach statistical significance (43). Both the PEACE-1 and ENZAMET analyses in elderly patients have been presented at conferences but data have not yet been published in full.

Subgroup analysis of the ARASENS data showed that the benefits of triplet *versus* ADT + docetaxel are directionally consistent in all age groups and ethnic/geographic/racial groups (12), including Chinese patients (56), North American patients (20), and Black/African-American patients (20, 21). Ongoing trials such as the PANTHER study are evaluating the effect of the combination of apalutamide + abiraterone + prednisone in clinical efficacy in Black men with mCRPC who are typically under-represented in clinical trials (64). Interim results indicate that Black participants had greater radiographic PFS, time to PSA progression, and overall survival than White participants (64). ARACOG is an ongoing, prospective, randomized, open-label phase II study comparing cognitive outcomes between men with metastatic and non-metastatic CRPC or mHSPC in the United States (65). Patients will be randomized (1:1) to receive treatment with enzalutamide 160 mg orally daily or darolutamide 600 mg orally twice daily, in combination with standard LHRH agonist-based treatment. Cognitive assessments will be to assess cognitive function and impairment (65).

#### 3.1.4 Results–patient-reported outcomes

To date, only the ARASENS study has reported patient-reported outcomes (PROs) data and only as conference abstracts (15, 18). These data suggest that the addition of an ARPI does not negatively impact on patient quality of life, with similar quality of life in the triplet and ARPI + docetaxel doublet therapy arms (15, 18).

#### 3.1.5 Results–toxicity

In the ARASENS study, the overall incidences of adverse events (AEs), serious AEs, grade ≥3 AEs and fatal AEs were similar in patients receiving ADT + docetaxel doublet *versus* triplet therapy; 98.5% and 99.5% of patients in these groups, respectively, developed any AEs, 42.3% and 44.8% experienced serious AEs, 63.5% and 66.1% had grade 3 or 4 AEs, and 4.0% and 4.1% died as a result of AEs (12). The highest incidence of AEs was seen with docetaxel and the most common AEs were those related to docetaxel in both groups, i.e., alopecia, fatigue, anemia and neutropenia (which included the preferred terms of leukopenia, neutropenia, decreased neutrophil count and decreased white blood cell count). Neutropenia occurred in 38.8% of patients in the doublet therapy arm and 39.3% in the triplet therapy arm, alopecia in 40.6% and 40.5%, respectively, fatigue in 32.9% and 33.1%, respectively, and anemia in 25.1% and 27.8%, respectively. AEs that occurred at a higher incidence in the group receiving darolutamide *versus* placebo were rash (16.6% vs. 13.5%) and hypertension (13.7% vs. 9.2%), although the rate of these events was similar in the two groups when adjusted for exposure time (rash: 6.2 vs. 7.3 per 100 patient-years [PY], respectively; hypertension: 4.9 per 100 PY in both groups) (12). AEs led to treatment discontinuation in 13.5% of patients in the darolutamide group and 10.6% of patients in the placebo group.

In the PEACE-1 study, 100% of patients receiving abiraterone-containing triplet therapy and 100% receiving ADT + docetaxel doublet therapy developed an AE of any severity, 63% *versus* 52% of patients who received triplet *versus* doublet therapy developed at least one grade ≥3 AE, and 2% *versus* 1%, respectively, developed a fatal AE (13). However, the incidence of severe AEs per 100 PY of treatment was 49 in the group receiving triplet therapy with abiraterone *versus* 55 in the group receiving doublet therapy. Abiraterone was stopped because of toxicity in 29 patients (21.0%) receiving triplet therapy and in one patient (<1.0%) receiving doublet therapy. AEs occurring at a higher incidence in the triplet *versus* ADT + docetaxel doublet arms of the PEACE-1 study, respectively, were hypertension (22% vs. 13%) and hepatotoxicity with elevated aminotransferase levels (6% vs. 1%) (13). Grade ≥3 neutropenia occurred at a similar rate in patients receiving triplet therapy and those receiving ADT + docetaxel (10% and 9%, respectively).

In the ENZAMET study, the overall incidence of AEs was similar in patients receiving enzalutamide *versus* a first-generation ARPI as part of triple therapy, but more patients in the first-generation ARPI group had grade 1–2 AEs (51% vs. 31%) and more patients in the enzalutamide group had grade 3 AEs (58% vs. 37%) (14). The most common grade 3–4 AEs were febrile neutropenia during docetaxel, which developed in 6% of patients in both treatment groups, fatigue (occurring in 1% of the control group vs. 5% of the enzalutamide group) and hypertension (occurring in 6% of the control group vs. 10% of the enzalutamide group) (14). Deaths due to serious AEs occurred in 10 patients (2%) in the control group and 13 patients (2%) in the enzalutamide group; no deaths were considered to be caused by enzalutamide.

It is expected that triplet therapy regimes would precipitate more AEs than doublet regimens. Overall, AE occurrence has been consistent among the three main RCTs and no new safety concerns have been identified.

### 3.2 Meta-analyses

Of the 14 meta-analyses identified, five compared triplet therapy with doublet therapy based on drug class groupings (48, 50, 56, 58, 59), and 11 used network meta-analysis (NMA) to compare regimens by individual ARPI (49, 51, 52, 53, 54, 55, 56, 57, 59, 60, 61).

#### 3.2.1 Survival outcomes in meta-analyses

The five meta-analyses that compared triplet with ADT + docetaxel doublet therapy reported a consistent overall improvement in OS with triplet therapy of about 25% (Table 3) (48, 50, 56, 58, 59).

**TABLE 3:** Effect of triplet therapy *versus* control on survival endpoints in meta-analyses.

Reference	No. of studies included	No. of patients included	Control	HR (95% CI)		
				OS	PFS	Radiographic PFS
Ciccarese 2022 (48)	5	NR	ADT + docetaxel	0.73 (0.65–0.83)	NR	NR
Fiorica 2022 (50)	5	2,538	ADT + docetaxel	0.74 (0.66–0.83)	0.41 (0.35–0.49)	NR
Maiorano 2022 (58)	5	2,836	ADT + docetaxel	0.74 (0.66–0.84)	0.49 (0.41–0.58)	0.50 (0.42–0.62)
Roy 2022 (59)	9	11,456	ADT + docetaxel	0.76 (0.64–0.91)	NR	NR
			ADT + ARPI^a^	0.89 (0.68–1.16)	NR	NR
Jian 2023 (56)	12	11,386	ADT ± SNA	0.57 (0.48–0.67)	NR	0.33 (0.26–0.41)

^a^ Indirect comparison based on network meta-analysis.

ADT, androgen deprivation therapy; ARPI, androgen receptor pathway inhibitor; CI, confidence interval; HR, hazard ratio; NR, not reported; OS, overall survival; PFS, progression-free survival; SNA, standard nonsteroidal antiandrogen.

#### 3.2.2 Survival outcomes in network meta-analyses

The 11 NMAs included between seven and 28 studies of triplet and doublet combinations conducted in between 5,804 and 12,298 patients (49, 51, 52, 53, 54, 55, 56, 57, 59, 60, 61). Because the NMAs included doublet therapy studies, the analyses also included apalutamide, which has not been studied as part of triplet therapy in RCTs. The survival analyses showed that darolutamide triplet therapy was consistently associated with a significant improvement in OS compared with ADT + docetaxel (Supplementary Figure S1A) (49, 52, 54, 55, 60). Abiraterone-containing triplet therapy was associated with a significant improvement in OS compared with ADT + docetaxel in three out of four NMAs analyzing this association (Supplementary Figure S1B) (49, 52, 55, 60), but neither enzalutamide- nor apalutamide-containing triplet regimens were associated with a significant improvement in OS compared with ADT + docetaxel (Supplementary Figures S1C, S1D) (49, 52, 54, 60, 61). The NMAs also evaluated indirect comparisons between triplet therapy and ADT + ARPI doublet therapy. In all cases, triplet therapy was not significantly more effective than ADT + ARPI doublet at improving OS (Supplementary Figures S2A–C) (53, 54, 55, 57, 60, 61), and in one case, abiraterone + ADT doublet therapy was found to be significantly better than abiraterone-based triplet therapy (Supplementary Figure S2C) (53).

The NMA data regarding PFS were generally similar to the OS data, with triplet therapy containing abiraterone or darolutamide being significantly better than SOC (ADT, ADT + ARPI or ADT + docetaxel) in one NMA (Supplementary Table S1) (53). Abiraterone-containing triplet therapy was significantly superior to ADT + docetaxel in two NMAs (55, 60). Enzalutamide-containing triplet therapy was superior to ADT + docetaxel in one NMA (60) but not in another (61). In most comparisons of triplet therapy with ARPI + ADT doublets, there was no significant differences in PFS (Supplementary Table S1), but Wang and colleagues found a significant improvement in PFS with abiraterone- or enzalutamide-containing triplet therapy compared with a doublet regimen of abiraterone + ADT (60). Triplet combinations were significantly superior to ADT + docetaxel for improving radiographic PFS and time to CRPC in an analysis by Jian and colleagues (Supplementary Table S2) (52).

When the data were analyzed by subgroup, none were significant in patients with low-volume disease (Supplementary Table S3), but some of the NMA comparisons of OS were statistically significant in patients with high-volume disease (Supplementary Table S4). With regard to PFS, none of the comparisons of abiraterone triplet therapy with any comparator (ADT alone, ADT + docetaxel or ADT + ARPI) were statistically significant in the low-volume disease groups (Supplementary Table S5) (55), whereas abiraterone was associated with a significant improvement in PFS in patients with high-volume disease compared with ADT alone (51, 54, 55), ADT + docetaxel (55), and ADT + apalutamide (55), but not compared with ADT + enzalutamide (55).

In the subgroup of patients with synchronous disease, significant improvements in OS were seen with triplet therapy compared with ADT alone or ADT + docetaxel, but not with ADT + ARPI (Supplementary Table S6) (55). No significant improvement was seen with triplet therapy vs. any comparator in patients with metachronous disease (Supplementary Table S6) (55).

#### 3.2.3 Toxicity

Eight meta-analyses evaluated the effects of triplet therapy on the incidence of AEs (49, 50, 52, 54, 56, 58, 60, 61), but the comparisons were mostly with ADT alone (54, 60, 61), ADT ± standard nonsteroidal antiandrogen (56) or docetaxel alone (49). The meta-analysis by Jian and colleagues was the only one to specifically compare AE rates during individual triplet regimens (abiraterone + ADT + docetaxel, and darolutamide + ADT + docetaxel) *versus* doublet therapy (52). In this analysis, both abiraterone- and darolutamide-containing triplet therapy were associated with a higher risk of hypertension compared with ADT + docetaxel, but only abiraterone + ADT + docetaxel was associated with a higher risk of grade ≥3 AEs (Supplementary Figure S3) (52). The risks of neutropenia and febrile neutropenia were similar with triplet regimens and ADT + docetaxel (52).

Menges and colleagues evaluated the net clinical benefit of doublet and triplet regimens, as the number of deaths avoided per 1,000 patients compared with ADT alone, weighted by 0.18 for incident grade 1–2 AEs and by 0.53 for incident grade 3–4 AEs (54). In this analysis, the probability of having a net clinical benefit over 24 months compared with ADT alone was 63.3%–78.7% for ARPI + ADT doublet regimens and 20.0% for darolutamide + ADT + docetaxel, and over 5 years was 66.7%–83.2% with ARPI + ADT doublet regimens and 23.5% for darolutamide + ADT + docetaxel. A sensitivity analysis using lower preference weights for AEs increased the probabilities, but did not change the relative rankings (54). No analysis was conducted comparing the net clinical benefit of triplet therapy with that of ARPI + ADT doublet therapy.

AE results data for the meta-analyses were similar to those obtained for RCTs.

### 3.3 Observational studies

There is also real-world evidence to support the use of triplet therapy in mHSPC. Our search identified one observational study in patients who received abiraterone (n = 77), darolutamide (n = 17), apalutamide (n = 2) or enzalutamide (n = 1) in combination with docetaxel and ADT (47). All the patients who received triplet therapy experienced a 99% decrease in PSA levels, and a radiologic response or stable disease was confirmed in 88% of those receiving abiraterone-based triplet therapy and 75% of those receiving darolutamide-based triplet therapy. In the abiraterone group, mean time to progression or CRPC was 8.5 months, time to second-line therapy was 8 months and time to death was 14.7 months. Abiraterone and darolutamide were associated with a similar incidence of AEs of any grade (62.3% and 58.8%, respectively) and grade ≥3 (19.5% and 11.8%). The most common AEs occurring during triplet therapy were fatigue, polyneuropathy and dermatologic conditions.

## 4 Discussion

The available RCT data and meta-analyses indicate that triplet therapy is often associated with better clinical outcomes compared with ADT + docetaxel, particularly for patients with synchronous, high-volume metastatic disease (5). Of note, most of the patients enrolled in ARASENS and PEACE-1 were at higher risk than usual (12, 13). To date, there is no randomized evidence on whether systemic triplet therapy improves outcomes compared with doublet therapy with ADT + an ARPI.

In the RCTs that allowed direct comparison of triplet therapy with ADT + docetaxel doublet therapy (ARASENS and PEACE-1), triplet therapy increased OS in the overall study populations by 32.5% and 25%, respectively (12, 13), and in meta-analyses the increase in OS was about 25%, with the HR ranging from 0.73 to 0.76 (48, 50, 56, 58, 59). It is possible that the magnitude of the OS improvement in the meta-analyses would have been greater if they had not included the ENZAMET study, which did not directly compare triplet therapy with ADT + docetaxel doublet therapy.

Both the timing and volume of metastases are independent prognostic indicators in mHSPC (66), and these parameters influence the magnitude of the benefit of triplet and doublet therapy. The available data indicate that patients with high-volume metastatic burden derive a significant benefit from triplet therapy, particularly those with synchronous mHSPC, but the benefit is less clear in patients with a low metastatic burden. Research has shown that, in patients with low-volume prostate cancer, metachronous disease has a more ADT-responsive transcriptional profile than synchronous disease (67), which may explain the better survival outcomes during ARPI therapy among those with synchronous *versus* metachronous disease.

The importance of both high-volume and synchronous metastases as determinants of response to triplet therapy is reflected in the latest iterations of major guidelines. The NCCN^®^ guidelines recommend triplet therapy (with abiraterone or darolutamide) or doublet ADT + ARPI therapy (with abiraterone, apalutamide or enzalutamide) as options for patients with high-volume synchronous metastases, high-volume metachronous metastases, or low-volume synchronous metastases, but recommend ADT + ARPI doublet therapy for patients with low-volume metachronous metastases (8). European Society for Medical Oncology (ESMO) guidelines recommend two triplet regimens as first-line therapy for mHSPC: abiraterone + ADT + docetaxel for fit men with synchronous (*de novo*) mHSPC (especially those with visceral or >3 bone metastases) or darolutamide + ADT + docetaxel for patients with synchronous or metachronous mHSPC (7). Similarly, other international guidelines recommend both triplet therapy (ADT + docetaxel + abiraterone or darolutamide) and doublet therapy (ADT + abiraterone) for patients with mHSPC (6). The only requirement for triplet therapy is that the patient is eligible to receive docetaxel (6). Further data are needed to determine whether triplet therapy may be superior to ADT + ARPI doublet therapy in patients with low-volume disease (e.g., younger patients with aggressive tumors or patients with high-risk disease) (68, 69).

ENZAMET (11) and PEACE-1 (13) did not include triplet therapy as a predefined treatment arm, and not all randomized patients in ENZAMET who received triplet therapy received a next-generation ARPI. Therefore, these studies were probably underpowered to detect significant differences among those who received triplet therapy regimens. Another potential confounder in the PEACE-1 study was that some patients also received radiotherapy to the primary tumor, and the data from the radiotherapy analysis have not yet been published. A preliminary analysis presented at the 2023 American Society of Clinical Oncology (ASCO) Annual Meeting suggested that outcomes were better in men receiving ADT (±docetaxel) + ARPI with radiotherapy compared with those receiving ADT (±docetaxel) alone, but the analysis did not compare outcomes among radiotherapy recipients who did *versus* did not receive docetaxel, so it is not clear how the use of radiotherapy may affect the outcomes in the triplet therapy arm of this study (16). Adding radiotherapy to ADT (±docetaxel) did not improve outcomes compared with ADT (±docetaxel) alone. Interestingly, prostate radiotherapy was associated with a reduction in serious genitourinary events regardless of metastatic burden.

All patients in the clinical trials of triplet therapy had an Eastern Cooperative Oncology Group performance status (ECOG PS) ≤2 and a median patient age of about 67 years, so there are no data on the use of triplet therapy in patients with more compromised performance status. The ENZAMET and PEACE-1 studies included patients with ECOG PS of 2. In subgroup analyses from these studies, the benefit of triplet therapy with a next-generation ARPI (abiraterone in PEACE-1 or enzalutamide in ENZAMET) on the primary endpoints was similar in patients with ECOG PS 1–2 and those with ECOG PS 0 (13, 14).

Patient fitness is a consideration when making any treatment decision in cancer. Real-world data show that outcomes are maximized in patients who can complete six cycles of docetaxel (70), so patients who develop docetaxel-related toxicity may not derive the optimal benefit of triplet therapy. In the ARASENS study, 87.6% of patients receiving darolutamide and 85.5% of those receiving placebo were able to complete six cycles of docetaxel (12), while in ENZAMET 65% of patients receiving enzalutamide triplet therapy completed six cycle of docetaxel (11). The PEACE-1 study did not report the proportion of patients who completed six cycles of docetaxel as part of an abiraterone-containing triplet regimen, but did note that the median number of cycles completed was six (13). Similarly high rates of docetaxel completion (86.7%) were reported in the real-world observational study of triplet therapy in routine clinical practice (47).

There are limited data on the effects of triplet therapy on PROs. Preliminary PRO data from ARASENS have been presented as a conference abstract, and indicated that HRQoL was similar in the triplet and doublet therapy arms (15, 18).

Finally, it is worth noting that, despite guideline recommendations, intensified therapy appears to be underutilized in the United States and Europe, even among patients who are candidates for these strategies (71). Therefore, many patients who could benefit from triplet therapy may not be receiving it, and the reasons for underutilization are not clearly known. Further, there is a need to individualize treatments considering patient-dependent factors such as age, comorbidities, clinical factors (disease presentation, risk, and volume) and the kinetics of PSA response to therapies; and to develop and validate biomarkers. The authors of the ENZAMET trial noted that the choice of new hormonal therapy will be reliant on its availability and on the patient’s age and comorbidity profile (14).

To date, we do not know which patients benefit most from triplet therapy, nor do we know in whom docetaxel can be omitted, nor whether such triplet therapy is superior to ADT + ARPI (72). Currently, no single triplet regimen can be considered superior to another because they have not been directly compared in a clinical trial. Moreover, a key limitation of the available RCT data is that the comparison doublet therapy was ADT + docetaxel, which was subsequently superseded by ADT + ARPI as the preferred doublet regimen. There have been no direct comparisons of triplet therapy with a doublet regimen comprising ADT + ARPI.

The available NMAs using indirect comparisons suggest that there is a statistically significant benefit with triplet therapy *versus* ADT + ARPI in patients with high-volume metastases (53, 54, 55, 57, 60, 61). While the NMA results are interesting, it is important to remember that they have their limitations. First, NMAs are subject to heterogeneity and incoherence, depending on the quality and quantity of the data supporting each direct and indirect comparison (73). Indirect comparisons assume transitivity, i.e., that if A = B and B=C then A = C (74). In fact, even when identical treatments are used in different studies, the magnitude of the effect may differ if the study populations are not identical (74). Heterogeneity between studies can lead to incoherence between the direct and indirect comparisons, which can be significant (75). Finally, NMAs are affected by publication bias and potentially by selection bias. Notwithstanding these limitations, in the absence of direct comparisons, NMAs are the only method for undertaking comprehensive comparisons of multiple interventions, overcoming the limitations of pairwise meta-analysis (76). Therefore, they provide valuable comparative effectiveness estimates that can be used for treatment decision-making (76).

## 5 Conclusion

Based on available data, triplet therapy appears to be the most effective first-line treatment regimen for men with mHSPC who have high-volume metastatic burden, particularly those with synchronous mHSPC. In patients with low-volume metachronous mHSPC, doublet therapy with an ADT + ARPI may be the most appropriate choice. For other patients with high- or low-risk disease, a darolutamide-based triplet therapy regimen may also be of benefit. To date, there are a lack of data to compare triplet therapy with ADT + ARPI doublet therapy. Further evidence is required to identify prognostic and predictive factors, beyond disease volume and metastatic burden, to make therapeutic decisions, and also to identify patients who can be spared docetaxel, without compromising survival outcomes.

## Abbreviations

ADT, androgen deprivation therapy (a GnRH agonist or GnRH antagonist or orchiectomy); AEs, adverse events; ALP, alkaline phosphatase; ARPI, androgen receptor pathway inhibitor; ASCO, American Society of Clinical Oncology; BID, twice daily; BMD, bone mineral density; CI, confidence interval; CRPC, castration-resistant prostate cancer; CYP, cytochrome P450; ECOG PS, Eastern Cooperative Oncology Group performance status; ESMO, European Society for Medical Oncology; GnRH, gonadotropin hormone-releasing hormone; HR, hazard ratio; mHSPC, metastatic hormone-sensitive prostate cancer; NA, not available; NMA, network meta-analysis; NR, not reported; OD, once daily; OS, overall survival; PFS, progression-free survival; PROs, patient-reported outcomes; PSA, prostate-specific antigen; PY, patient-year; QoL, quality of life; RCT, randomized controlled trial; RT, radiotherapy; SNA, standard nonsteroidal antiandrogen; SOC, standard of care.
