# Supplementary material for: Triplet systemic therapy for hormone-sensitive prostate cancer: a critical review with a multidisciplinary approach
Source: Oncol Rev. 2025 Jul 25;19:1599292. doi: 10.3389/or.2025.1599292 (PMC12331671; doi:10.3389/or.2025.1599292)
Supplement: Supplementary file 1 [file DataSheet1.pdf]

Supplementary Material

1 Supplementary Data

1.1 Search strategies

1.1.1 PubMed

Step	Query
#1	((“castration sensitive”[Text Word] OR “castrate sensitive”[Text Word] OR “hormone sensitive”[Text Word]) AND (“prostate”[Text Word] OR “prostatic”) AND (“metast*”[Text Word] OR “oligometast*”[Text Word])) OR (mHSPC[Text Word] OR mCSPC[Text Word])
#2	(“Docetaxel”[Mesh] OR Docetaxel*[Text Word] OR AXTERE*[Text Word] OR TAXANIT*[Text Word] OR DOCEFREZ*[Text Word] OR Taxotere[Text Word] OR Docetaxol[Text Word] OR Taxoltere[Text Word] OR DoceAqualip*[Text Word] OR Nanoxel*[Text Word] OR “RP 56976” [Text Word] OR RP-56976”[Text Word] OR RP56976[Text Word])
#3	(“Androgen antagonists[Mesh]) OR “androgen deprivation therapy”[Text Word] OR “androgen deprivation treatment”[Text Word] OR ADT[Text Word] OR “Primary hormone therapy”[Text Word] OR “androgen ablation therapy” [Text Word] OR “androgen suppression therapy[Text Word] OR “Anti androgen*” [Text Word] OR Anti-androgen*[Text Word] OR Antiandrogen[Text Word] OR “Androgen antagonist” [Text Word])
#4	(“Goserelin”[Mesh])) OR (goserelin[Text Word] OR “Zoladex*”[Text Word] OR “ICI 118630”[Text Word] OR “ICI-118630”[Text Word] OR “ICI118630”[Text Word] OR “ZD 9393”[Text Word] OR “ZD-9393”[Text Word] OR “ZD9393”[Text Word])
#5	(“Leuprolide”[Mesh) OR (leuprolide[Text Word] OR “Camcevi*”[Text Word] OR “Leuprorelin”[Text Word] “Enantone”[Text Word] OR “Lupron”[Text Word] OR “TAP 144”[Text Word] OR “TAP-144”[Text Word] OR “TAP144”[Text Word] OR “A 43818”[Text Word] OR “A-43818”[Text Word] OR “A43818”[Text Word] OR “FP 001”[Text Word] OR “FP-001”[Text Word] OR “FP-001 LMIS”[Text Word] OR “FP-001/LMIS”[Text Word] OR “FP001”[Text Word] OR “LMIS”[Text Word])
#6	(“Triptorelin pamoate”[Mesh) OR (triptorelin[Text Word] OR “Pamorelin*”[Text Word] OR “Trelstar*”[Text Word] “Decapeptyl”[Text Word] OR “CL 118532”[Text Word] OR “CL-118532”[Text Word] OR “CL118532”[Text Word] OR “Wy 42462”[Text Word] OR “Wy-42462”[Text Word] OR “Wy42462”[Text Word] OR “AY 25650”[Text Word] OR “AY-25650”[Text Word] OR “AY25650”[Text Word])
#7	(“acetyl-2-naphthylalanyl-3-chlorophenylalanyl-1-oxohexadecyl-seryl-4-aminophenylalanyl(hydroorotyl)-4-aminophenylanalyl(carbamoyl)-leucyl-lLys-prolyl-alaninamide”[Supplementary Concept]) OR (degarelix[Text Word] OR “Gonax*”[Text Word] OR “Firmagon*”[Text Word] OR “ASP 3550” [Text Word] OR “ASP-3550” [Text Word] OR “ASP3550” [Text Word] OR “FE 200486” [Text Word] OR “FE-200486” [Text Word] OR “FE200486” [Text Word])
#8	(“relugolix”[Supplementary Concept]) OR “relugolix”[Text Word] or “ORGOVYX*”[Text Word] OR “MVT 601*”[Text Word] OR “MVT-601*”[Text Word] OR “MVT601*”[Text Word])
#9	#1 AND (#2 AND (#3 OR #4 OR #5 OR #6 OR #7 OR #8))

#10	(“Abiraterone acetate”[Mesh]) OR (abiraterone[Text Word] OR “Zytiga*”[Text Word] OR “Zitiga*”[Text Word] “Zaytiga*”[Text Word] OR “Yonsa*”[Text Word] OR “AAFP”[Text Word] OR “Aviraterone acetate”[Text Word] OR “CB 7630”[Text Word] OR “CB-7630”[Text Word] OR “CB7630”[Text Word] OR “JNJ 212082”[Text Word] OR “JNJ-212082”[Text Word] OR “JNJ212082”[Text Word])
#11	(“apalutamide”[Supplementary Concept]) OR (apalutamide[Text Word] OR “ERLEADA*”[Text Word] OR “ERLYAND*”[Text Word] OR “ARN 509”[Text Word] OR “ARN-509”[Text Word] OR “ARN509”[Text Word] OR “JNJ 56021927”[Text Word] OR “JNJ-56021927”[Text Word] OR “JNJ560219217”[Text Word] OR “JNJ 927”[Text Word] OR “JNJ-927”[Text Word] OR “JNJ927”[Text Word])
#12	(“darolutamide”[Supplementary Concept]) OR (darolutamide[Text Word] OR “DARO”[Text Word] OR “Darramamide”[Text Word] “NUBEQA*”[Text Word] OR “ODM 201”[Text Word] OR “ODM-201”[Text Word] OR “ODM201”[Text Word] OR “BAY 1841788”[Text Word] OR “BAY-1841788”[Text Word] OR “BAY1841788”[Text Word])
#13	(“enzalutamide”[Supplementary Concept]) OR (enzalutamide[Text Word] OR “Xtandi*”[Text Word] OR “ASP 9785”[Text Word] OR “ASP-9785”[Text Word] OR “ASP9785”[Text Word] OR “MDV 3100”[Text Word] OR “MDV-3100”[Text Word] OR “MDV3100”[Text Word] OR “HC 1119”[Text Word] OR “HC-1119”[Text Word] OR “HC1119”[Text Word])
#14	#9 AND (#10 OR #11 OR #12 OR #13)
#15	(triplet[Text Word] OR triple[Text Word]) AND (therapy[Text Word] OR treatment[Text Word]) OR regimen[Text Word] OR combin*[Text Word])
#16	(#1 AND #2) AND #15
#17	#14 OR #16
#18	((Review[Publication type] OR (Case reports[Publication Type])) OR (News[Publication Type])
#19	retrospective*[Title]
#20	animal*[Title] OR rat[Title] OR rats[Title] OR mice[Title] OR mouse[Title] OR murine*[Title] OR “guinea pig*”[Title] OR rabbit*[Title] OR monkey*[Title] OR dog[Title] dogs[Title] OR canine*[Title] OR cat[Title] OR cats[Title] OR feline*[Title] OR pig[Title] OR pigs[Title] OR sheep[Title] OR “in vitro”[Title] OR in-vitro[Title] OR invitro[Title] OR “cell* culture*”[Title] OR cell-culture*[Title] OR cellculture*[Title] OR “cell* line*”[Title] OR cell-line*[Title] OR culture*[Title] OR germline[Title] OR germ-line[Title]
#21	#17 NOT (#18 OR #19 OR #20)
#22	#21; Filters: English

1.1.2 OVID Medline

Step	Query
#1	((“castration sensitive” OR “castrate sensitive” OR “hormone sensitive”) AND (“prostate” OR “prostatic”) AND (“metast*” OR “oligometastas*”)) OR (mHSPC OR mCSPC)).mp.
#2	Exp Docetaxel/ OR (Docetaxel* OR AXTERE* OR TAXANIT* OR TAXOCCORD* OR DOCEFREZ* OR Taxotere OR Docetaxol OR Taxoltere OR DoceAqualip* OR Nanoxel* OR “RP 56976” OR “RP-56976” OR RP56976).mp.
#3	Exp Androgen antagonists/ OR (“androgen deprivation therapy” OR “androgen deprivation treatment” OR ADT OR “Primary hormone therapy” OR “hormone therapy” OR “androgen ablation therapy” OR “androgen suppression therapy” OR “Anti androgen*” OR Anti-androgen* OR Antiandrogen OR “Androgen antagonist*”).mp.
#4	Exp Goserelin/ OR (goserelin OR Zoladex* OR “ICI 118630” OR “ICI-118630” OR “ICI118630” OR “ZD 9393” OR “ZD-9393” OR “ZD9393”).mp.

#5	Exp Leuprolide/ OR (leuprolide OR Camcevi* OR Leuprorelin OR Enantone OR Lupron OR “TAP 144” OR TAP-144 OR TAP144 OR “A 43818” OR A-43818 OR A43818 OR “FP 001” OR “FP-001” OR “FP-001 LMIS” OR “FP-001/LMIS” OR FP001 OR LMIS).mp.
#6	Exp Triptorelin pamoate/ OR (triptorelin OR Pamorelin* OR Trelstar* OR Decapeptyl OR “CL 118532” OR “CL-118532” OR “CL118532” OR “Wy 42462” OR “Wy-42462” OR “Wy42462” OR “AY 25650” OR “AY-25650” OR “AY25650”).mp.
#7	(degarelix OR Gonax* OR Firmagon* OR “ASP 3550” OR “ASP-3550” OR “ASP3550”).mp.
#8	(relugolix OR ORGOVYX* OR “MVT 601*” OR “MVT-601*” OR MVT601*).mp.
#9	1 AND (2 AND (3 OR 4 OR 5 OR 6 OR 7 OR 8)
#10	Exp Abiraterone acetate/ OR (abiraterone OR Zytiga* OR Zitiga* OR Zaitiga* OR Zaytiga* OR Yonsa* OR AAFP OR “Aviraterone acetate” OR “CB 7630” OR “CB-7630” OR CB7630 OR “JNJ 212082” OR “JNJ-212082” OR JNJ212082).mp.
#11	(apalutamide OR ERLEADA* OR ERLYAND* OR “ARN 509” OR “ARN-509” OR ARN509 OR “JNJ 56021927” OR “JNJ-56021927” OR JNJ560219217 OR “JNJ 927” OR “JNJ-927” OR JNJ927).mp.
#12	(darolutamide OR DARO OR Darramamide OR NUBEQA* OR “ODM 201” OR “ODM-201” OR ODM201 OR “BAY 1841788” OR “BAY-1841788” OR BAY1841788).mp.
#13	(enzalutamide OR Xtandi* OR “ASP 9785” OR “ASP-9785” OR ASP9785 OR “MDV 3100” OR “MDV-3100” OR MDV3100 OR “HC 1119” OR “HC-1119” OR HC1119).mp.
#14	9 AND (10 OR 11 OR 12 OR 13)
#15	(triplet OR triple) AND (therapy OR treatment OR regimen OR combin*).ti,ab,kw.
#16	1 AND 2 AND 15
#17	14 OR 16
#18	(Review OR Case reports OR News).pt. OR retrospective*.ti.
#19	animal* OR rat OR rats OR mice OR mouse OR murine* OR “guinea pig*” OR “rabbit*” OR monkey* OR dog OR dogs OR canine* OR cat OR cats OR feline* OR pig OR pigs OR sheep OR “in vitro” OR in-vitro OR invitro OR “cell* culture*” OR cell-culture* OR cellculture* OR “cell*line*” OR cell-line* OR culture* OR germline OR germ-line.ti.
#20	17 NOT (18 OR 19)
#21	Limit 20 to English language
#22	Remove duplications from 21

1.1.3 Web of Science

#1	((“castration sensitive” OR “castrate sensitive” OR “hormone sensitive”) AND (“prostate” OR “prostatic”) AND (“metast*” OR “oligometastas*”)) OR (mHSPC OR mCSPC))
#2	TS=(Docetaxel* OR AXTERE* OR TAXANIT* OR TAXOCCORD* OR DOCEFREZ* OR Taxotere OR Docetaxol OR Taxoltere OR DoceAqualip* OR Nanoxel* OR “RP 56976” OR “RP-56976” OR RP56976)
#3	#1 AND #2
#4	TS=(Abiraterone OR Zytiga* OR Zitiga* OR Zaitiga* OR Zaytiga* OR Yonsa* OR AAFP OR “Aviraterone acetate” OR “CB 7630” OR “CB-7630” OR CB7630 OR “JNJ 212082” OR “JNJ-212082” OR JNJ212082 OR apalutamide OR ERLEADA* OR ERLYAND* OR “ARN 509” OR “ARN-509” OR ARN509 OR “JNJ 56021927” OR “JNJ-56021927” OR JNJ560219217 OR “JNJ 927” OR “JNJ-927” OR JNJ927 OR darolutamide OR DARO OR Darramamide OR NUBEQA* OR “ODM 201” OR “ODM-201” OR ODM201 OR “BAY 1841788” OR “BAY-1841788” OR BAY1841788)
#5	#3 AND #4
#6	(TS=(((triplet OR triple) AND (therapy OR treatment OR regimen OR combin*))) AND TS=(Docetaxel* OR AXTERE* OR TAXANIT* OR TAXOCCORD* OR DOCEFREZ*

	OR Taxotere OR Docetaxol OR Taxoltere OR DoceAqualip* OR Nanoxel* OR “RP 56976” OR “RP-56976” OR RP56976))
#7	#1 AND #6
#8	#5 OR #7
#9	#5 OR #7 AND Review Article (Exclude – Document Types)
#10	TI=(“Case report” OR retrospective)
#11	#9 NOT #10
#12	#9 NOT #10 and English (Languages)

1.1.4 Cochrane Central Trials

Step	Query
#1	((“castration sensitive” OR “castrate sensitive” OR “hormone sensitive”) AND (“prostate” OR “prostatic”) AND (“metast*” OR “oligometastas*”)) OR (mHSPC OR mCSPC)).
#2	(Docetaxel* OR AXTERE* OR TAXANIT* OR TAXOCCORD* OR DOCEFREZ* OR Taxotere OR Docetaxol OR Taxoltere OR DoceAqualip* OR Nanoxel* OR “RP 56976” OR “RP-56976” OR RP56976)
#3	(“androgen deprivation therapy” OR “androgen deprivation treatment” OR ADT OR “Primary hormone therapy” OR “hormone therapy” OR “androgen ablation therapy” OR “androgen suppression therapy” OR “Anti androgen*” OR Anti-androgen* OR Antiandrogen OR “Androgen antagonist*”)
#4	MeSH descriptor: [Androgen Antagonists] explode all trees
#5	(goserelin OR Zoladex* OR “ICI 118630” OR “ICI-118630” OR “ICI118630” OR “ZD 9393” OR “ZD-9393” OR “ZD9393”)
#6	(leuprolide OR Camcevi* OR Leuprorelin OR Enantone OR Lupron OR “TAP 144” OR TAP-144 OR TAP144 OR “A 43818” OR A-43818 OR A43818 OR “FP 001” OR “FP-001” OR “FP-001 LMIS” OR “FP-001/LMIS” OR FP001 OR LMIS)
#7	(triptorelin OR Pamorelin* OR Trelstar* OR Decapeptyl OR “CL 118532” OR “CL-118532” OR “CL118532” OR “Wy 42462” OR “Wy-42462” OR “Wy42462” OR “AY 25650” OR “AY-25650” OR “AY25650”)
#8	(degarelix OR Gonax* OR Firmagon* OR “ASP 3550” OR “ASP-3550” OR “ASP3550”)
#9	(relugolix OR ORGOVYX* OR “MVT 601*” OR “MVT-601*” OR MVT601*)
#10	#1 AND #2
#11	#10 AND #3
#12	#10 AND #4
#13	#10 AND #5
#14	#10 AND #6
#15	#10 AND #7
#16	#10 AND #8
#17	#10 AND #9
#18	#10 OR #11 OR #12 OR #13 OR #14 OR #16
#19	(abiraterone OR Zytiga* OR Zitiga* OR Zaitiga* OR Zaytiga* OR Yonsa* OR AAFP OR “Aviraterone acetate” OR “CB 7630” OR “CB-7630” OR CB7630 OR “JNJ 212082” OR “JNJ-212082” OR JNJ212082 OR apalutamide OR ERLEADA* OR ERLYAND* OR “ARN 509” OR “ARN-509” OR ARN509 OR “JNJ 56021927” OR “JNJ-56021927” OR JNJ560219217 OR “JNJ 927” OR “JNJ-927” OR JNJ927 OR darolutamide OR DARO OR Darramamide OR NUBEQA* OR “ODM 201” OR “ODM-201” OR ODM201 OR “BAY 1841788” OR “BAY-1841788” OR BAY1841788 OR enzalutamide OR Xtandi* OR “ASP

	9785" OR "ASP-9785" OR ASP9785 OR "MDV 3100" OR "MDV-3100" OR MDV3100 OR "HC 1119" OR "HC-1119" OR HC1119)
#20	#18 AND #19
#21	((triplet OR triple) AND (therapy OR treatment OR regimen OR combin*))
#22	#10 AND #21
#23	#20 OR #22
#24	"case report".ti OR "retrospective".ti
#25	#23 NOT #24

2 Supplementary Tables

2.1 **Supplementary Table S1.** Progression-free survival reported in network meta-analyses.

References	HR (95% CI)		
	**Abiraterone triplet**	**Enzalutamide triplet**	**Darolutamide triplet**
**Compared with standard of care[a]**			
Lee 2023 (1)	0.50 (0.35–0.72)	NA	0.36 (0.30–0.43)
**Compared with ADT + docetaxel**			
Riaz 2023 (2)	0.50 (0.35–0.72)	NA	NA
Wang 2023 (3)	0.50 (0.38–0.65)	0.49 (0.37–0.64)	NA
Wang 2024 (4)	NA	0.51 (0.19–1.34)	NA
**Compared with ADT + ARPI**			
**ADT + abiraterone**			
Lee 2023 (1)	1.16 (0.84–1.61)	NA	NA
Wang 2023 (3)	0.72 (0.52–0.99)	0.71 (0.51–0.97)	NA
Wang 2024 (4)	0.77 (0.14–4.17)	NA	NA
**ADT + apalutamide**			
Riaz 2023 (2)	0.72 (0.45–1.08)	NA	NA
Wang 2023 (3)	0.69 (0.47–1.03)	0.68 (0.45–1.01)	NA
Wang 2024 (4)	NA	0.77 (0.15–4.06)	NA
**ADT + enzalutamide**			
Riaz 2023 (2)	0.88 (0.56–1.39)	NA	NA
Wang 2023 (3)	0.94 (0.65–1.36)	0.95 (0.64–1.34)	NA
Wang 2024 (4)	NA	0.70 (0.15–3.16)	NA
**Comparator is enzalutamide + ADT + docetaxel triplet regimen**			
Wang 2023 (3)	1.02 (0.70–1.48)	NA	NA

[a]Standard of care was ADT alone, ADT + docetaxel or ADT + ARPI.
ADT, androgen deprivation therapy; ARPI, androgen receptor pathway inhibitor; CI, confidence interval; HR, hazard ratio; NA, not analyzed.

2.2 **Supplementary Table S2.** Radiographic progression-free survival and time to castration-resistance prostate cancer in reported in the network meta-analysis by Jian and colleagues (5).

**Endpoint**	**HR (95% credible intervals)**			
	**Abiraterone triplet**	**Enzalutamide triplet**	**Apalutamide triplet**	**Darolutamide triplet**
**Compared with ADT + docetaxel**				
rPFS	0.49 (0.39–0.61)	0.52 (0.30–0.89)	0.47 (0.22–1.00)	NA
Time to CRPC	0.38 (0.31–0.47)	0.41 (0.25–0.67)	NA	0.35 (0.30–0.42)

ADT, androgen deprivation therapy; CRPC, castration-resistant prostate cancer; HR, hazard ratio; NA, not analyzed; rPFS, radiographic progression-free survival.

2.3 **Supplementary Table S3.** Overall survival in patients with low-volume disease reported in network meta-analyses.

References	HR (95% CI)		
	**Abiraterone triplet**	**Enzalutamide triplet**	**Darolutamide triplet**
**Compared with standard of care[a]**			
Lee 2023 (1)	0.83 (0.50–1.38)	NA	NA
**Compared with ADT + docetaxel**			
Riaz 2023 (2)	0.83 (0.50–1.83)	NA	NA
Wang 2023 (3)	0.83 (0.40–1.75)	NA	NA
**Compared with ADT + ARPI**			
**ADT + any ARPI**			
Hoeh 2023 (6)	1.27 (0.70–2.28)	NA	1.04 (0.58–1.87)
**ADT + abiraterone**			
Lee 2023 (1)	1.15 (0.59–2.24)	NA	NA
Wang 2023 (3)	1.12 (0.43–2.95)	0.87 (0.25–3.13)	NA
**ADT + apalutamide**			
Riaz 2023 (2)	1.45 (0.73–2.89)	NA	NA
Wang 2023 (3)	1.47 (0.50–4.34)	1.15 (0.30–4.40)	NA
**ADT + enzalutamide**			
Riaz 2023 (2)	1.14 (0.56–2.32)	NA	NA
Wang 2023 (3)	1.42 (0.54–4.06)	1.11 (0.32–4.20)	NA

[a]Standard of care was ADT alone, ADT + docetaxel or ADT + ARPI.
ADT, androgen deprivation therapy; ARPI, androgen receptor pathway inhibitor; CI, confidence interval; HR, hazard ratio; NA, not analyzed.

2.4 **Supplementary Table S4.** Overall survival in patients with high-volume disease reported in network meta-analyses.

References	HR (95% CI)			
	**Any ARPI triplet**	**Abiraterone triplet**	**Enzalutamide triplet**	**Darolutamide triplet**
**Compared with standard of care**[a]				
Lee 2023 (1)	NA	0.72 (0.55–0.95)	NA	NA
**Compared with ADT + docetaxel**				
Riaz 2023 (2)	0.72 (0.55–0.95)	0.72 (0.55–0.95)	NA	NA
Wang 2023 (3)	NA	0.72 (0.49–1.08)	0.97 (0.59–1.61)	NA
**Compared with ADT + ARPI**				
**ADT + any ARPI**				
Hoeh 2023 (6)	NA	0.79 (0.57–1.10)	NA	0.76 (0.59–0.67)
Riaz 2023 (2)	0.57 (0.44–0.75)	NA	NA	NA
**ADT + abiraterone**				
Lee 2023 (1)	NA	1.16 (0.84–1.61)	NA	NA
Wang 2023 (3)	NA	0.80 (0.47–1.38)	0.75 (0.39–1.40)	NA
**ADT + apalutamide**				
Riaz 2023 (2)	NA	0.75 (0.51–1.09)	NA	NA
Wang 2023 (3)	NA	0.75 (0.42–1.34)	1.01 (0.52–0.99[b])	NA
**ADT + enzalutamide**				
Riaz 2023 (2)	NA	0.79 (0.54–1.17)	NA	NA
Wang 2023 (3)	NA	0.85 (0.51–1.46)	1.08 (0.58–2.02)	NA

[a]Standard of care was ADT alone, ADT + docetaxel or ADT + ARPI; [b]As reported in the published paper.

ADT, androgen deprivation therapy; ARPI, androgen receptor pathway inhibitor; CI, confidence interval; HR, hazard ratio; NA, not analyzed.

2.5 **Supplementary Table S5.** Progression-free survival in patients with low- or high-volume disease reported in a network meta-analysis (2).

Comparator	HR (95% CI)	
	**Abiraterone triplet in LVD**	**Abiraterone triplet in HVD**
ADT alone	0.73 (0.43–1.31)[a]	0.52 (0.38–0.71)[a]
ADT + docetaxel	0.58 (0.29–1.15)	0.47 (0.30–0.73)
ADT + ARPI		
ADT + apalutamide	1.20 (0.51–2.83)	0.54 (0.32–0.90)
ADT + enzalutamide	1.72 (0.68–4.38)	0.66 (0.39–1.13)

[a]Identical results were obtained for these comparisons in two other network meta-analyses, by Hoeh and colleagues (6) and Mandel and colleagues (2023) (7).

ADT, androgen deprivation therapy; ARPI, androgen receptor pathway inhibitor; CI, confidence interval; HR, hazard ratio; HVD, high-volume disease; LVD, low-volume disease.

2.6 **Supplementary Table S6.** Overall survival in patients with metachronous versus synchronous disease reported in a network meta-analysis (2).

**Comparator**	**HR (95% CI)**		
	**Synchronous disease**		**Metachronous disease**
	**Abiraterone triplet**	**Darolutamide triplet**	**Darolutamide triplet**
ADT alone	0.57 (0.42–0.77)	0.54 (0.42–0.69)	0.55 (0.28–1.08)
ADT + docetaxel	0.75 (0.59–0.95)	0.71 (0.59–0.85)	0.61 (0.35–1.05)
ADT + ARPI			
ADT + apalutamide	0.84 (0.55–1.22)	0.79 (0.57–1.11)	1.41 (0.58–3.45)
ADT + enzalutamide	0.91 (0.62–1.32)	0.86 (0.61–1.21)	0.77 (0.33–1.83)

ADT, androgen deprivation therapy; ARPI, androgen receptor pathway inhibitor; CI, confidence interval; HR, hazard ratio.

3 Supplementary Figures

(A) Darolutamide

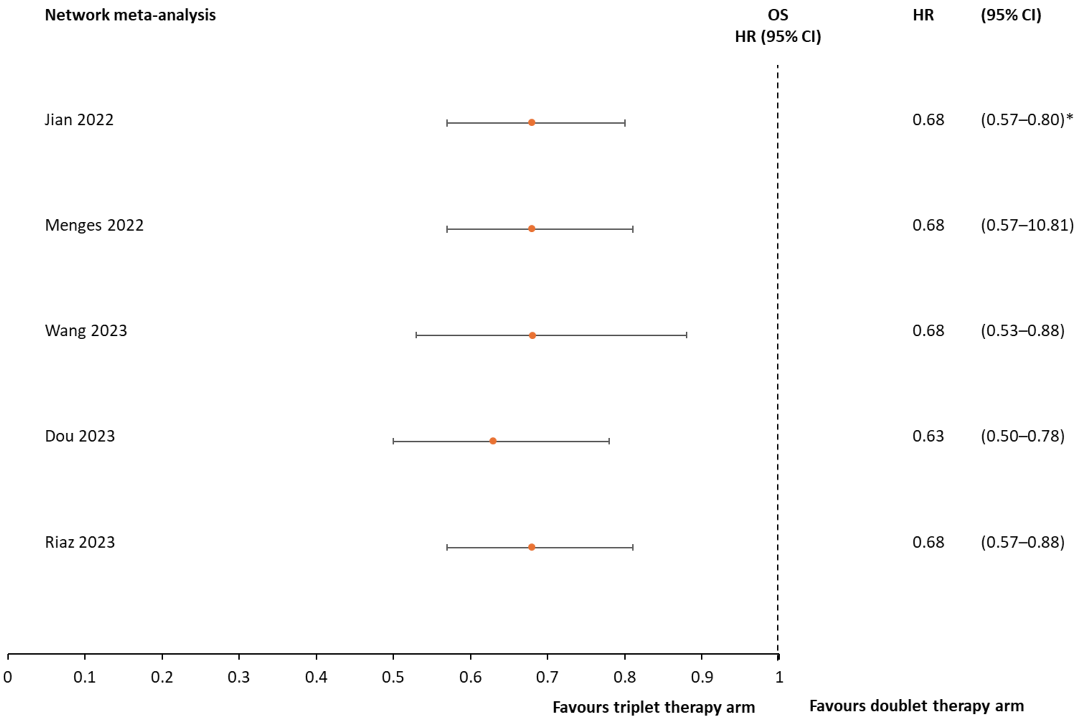

(B) Abiraterone

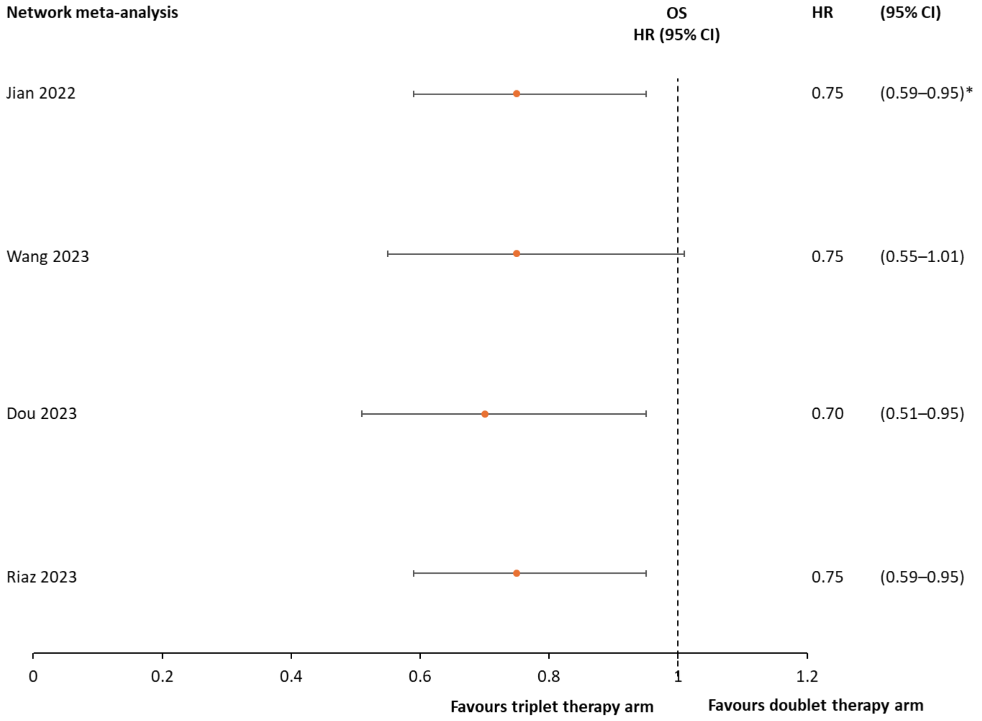

(C) Enzalutamide

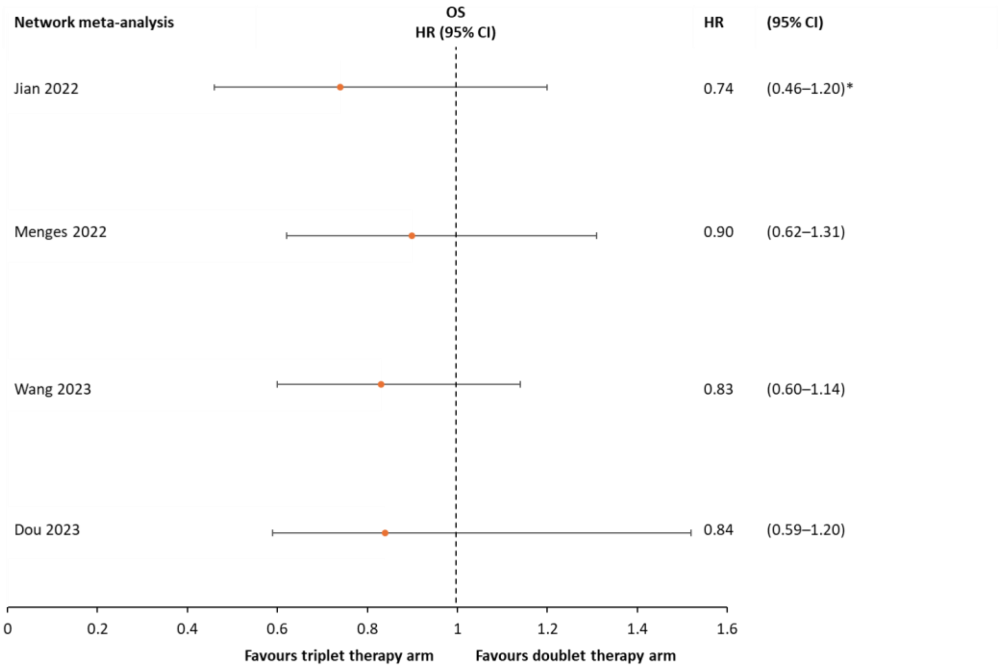

(D) Apalutamide

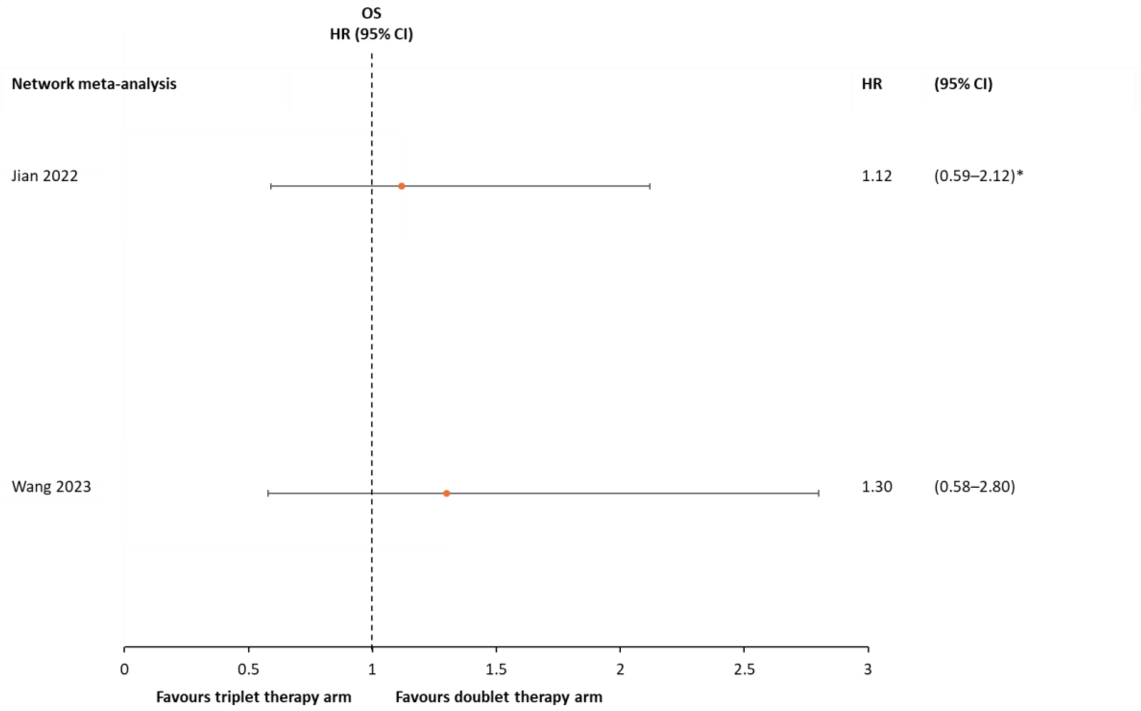

Supplementary Figure 1. Results of network meta-analyses examining the effects of triplet therapy versus androgen deprivation therapy + docetaxel doublet therapy on overall survival in men with metastatic hormone-sensitive prostate cancer, when the androgen receptor pathway inhibitor in the triplet therapy regimen is (A) darolutamide (2, 3, 5, 8, 9), (B) abiraterone (3, 5, 8, 9), (C) enzalutamide (3, 5, 8, 9) and (D) apalutamide (3, 5). *95% credible intervals reported in this study. CI, confidence interval; HR, hazard ratio; OS, overall survival.

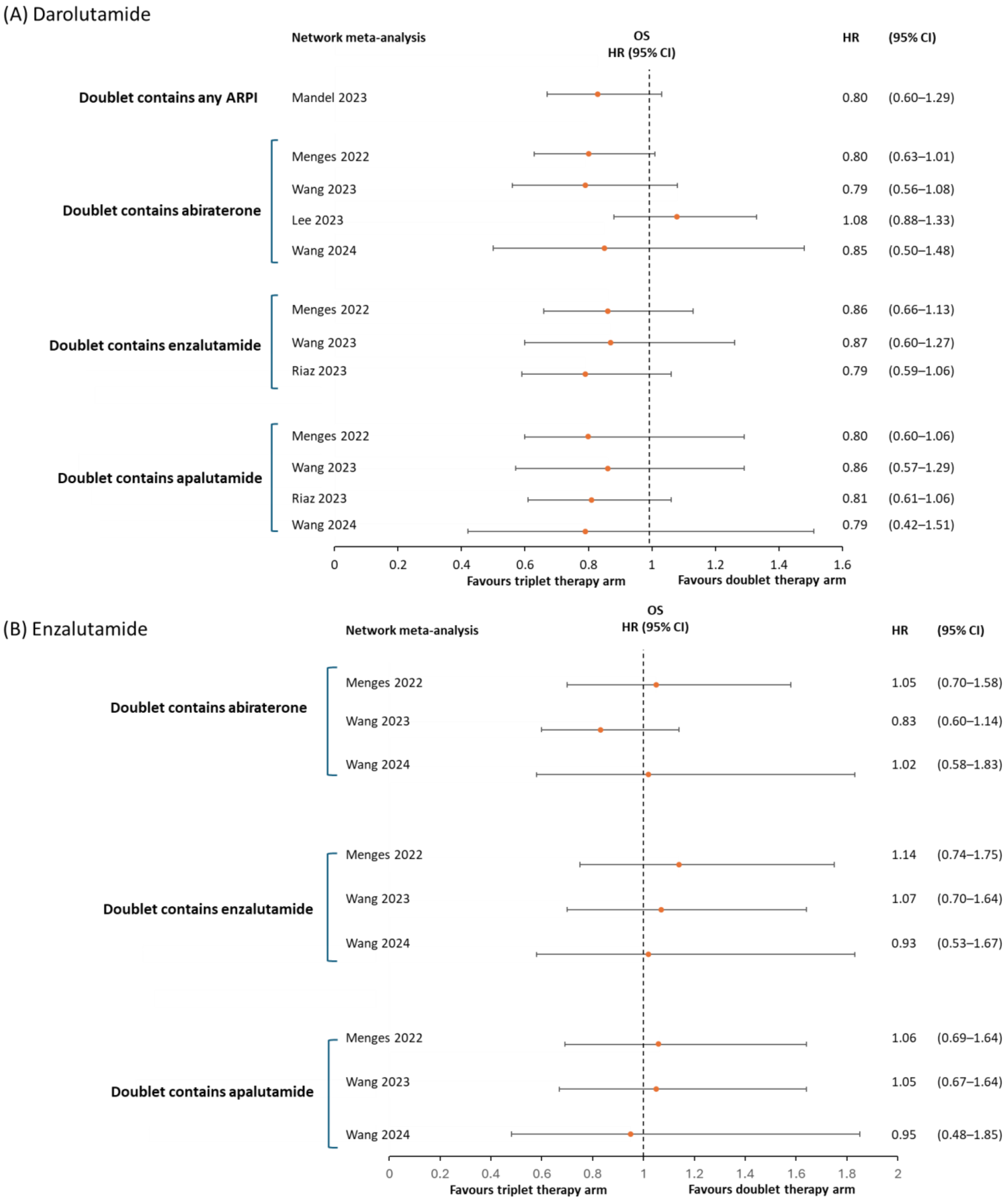
(A) Darolutamide
Network meta-analysis
OS
HR (95% CI)
HR
(95% CI)
Doublet contains any ARPI
Mandel 2023
0.80
(0.60–1.29)
Doublet contains abiraterone
Menges 2022
0.80
(0.63–1.01)
Wang 2023
0.79
(0.56–1.08)
Lee 2023
1.08
(0.88–1.33)
Wang 2024
0.85
(0.50–1.48)
Doublet contains enzalutamide
Menges 2022
0.86
(0.66–1.13)
Wang 2023
0.87
(0.60–1.27)
Riaz 2023
0.79
(0.59–1.06)
Doublet contains apalutamide
Menges 2022
0.80
(0.60–1.06)
Wang 2023
0.86
(0.57–1.29)
Riaz 2023
0.81
(0.61–1.06)
Wang 2024
0.79
(0.42–1.51)
0
0.2
0.4
0.6
0.8
1
1.2
1.4
1.6
Favours triplet therapy arm
Favours doublet therapy arm
(B) Enzalutamide
Network meta-analysis
OS
HR (95% CI)
HR
(95% CI)
Doublet contains abiraterone
Menges 2022
1.05
(0.70–1.58)
Wang 2023
0.83
(0.60–1.14)
Wang 2024
1.02
(0.58–1.83)
Doublet contains enzalutamide
Menges 2022
1.14
(0.74–1.75)
Wang 2023
1.07
(0.70–1.64)
Wang 2024
0.93
(0.53–1.67)
Doublet contains apalutamide
Menges 2022
1.06
(0.69–1.64)
Wang 2023
1.05
(0.67–1.64)
Wang 2024
0.95
(0.48–1.85)
0
0.2
0.4
0.6
0.8
1
1.2
1.4
1.6
1.8
2
Favours triplet therapy arm
Favours doublet therapy arm

(C) Abiraterone

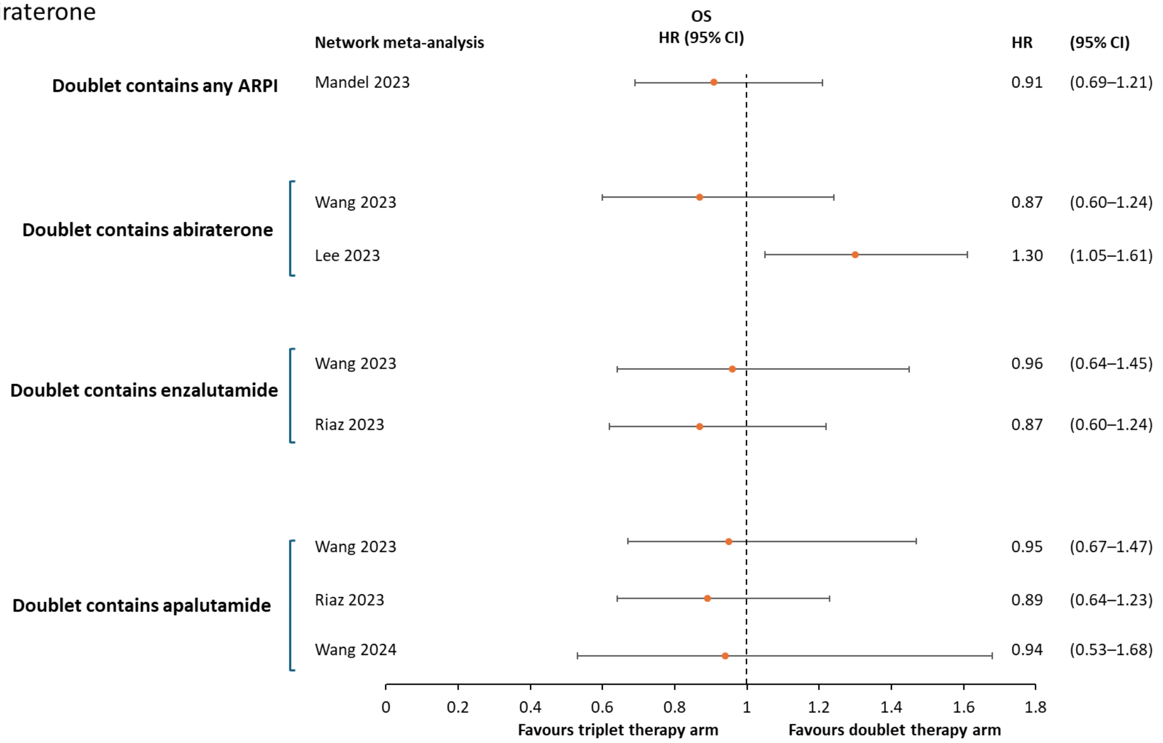

Supplementary Figure 2. Results of network meta-analyses examining the effects of triplet therapy versus androgen deprivation therapy + androgen receptor pathway inhibitor doublet therapy on overall survival in men with metastatic hormone-sensitive prostate cancer, when the androgen receptor pathway inhibitor in the triplet therapy regimen is (A) darolutamide, (B) enzalutamide and (C) abiraterone (1-4, 7, 9). ARPI, androgen receptor pathway inhibitor; CI, confidence interval; HR, hazard ratio; OS, overall survival.

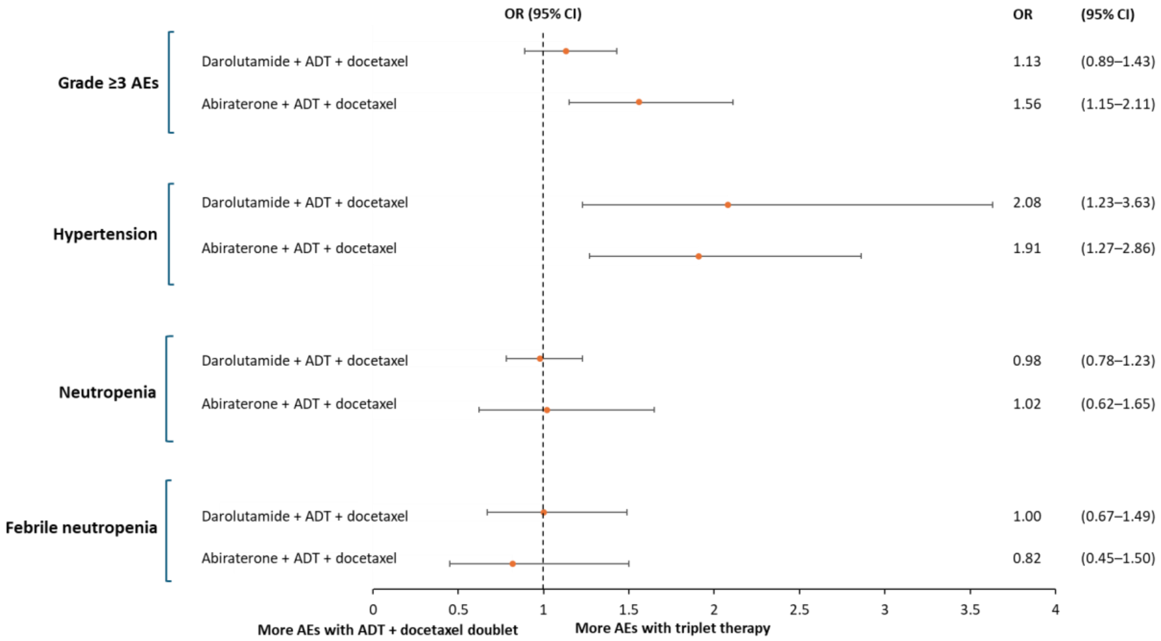

Supplementary Figure 3. Results of a network meta-analysis examining the effects of triplet therapy versus androgen deprivation therapy + docetaxel doublet therapy on adverse events (5). ADT, androgen deprivation therapy; AE, adverse event; CI, confidence interval; OR, odds ratio.

4 References

1. Lee YS, Kim SH, Tae JH, Chang IH, Kim TH, Myung SC, et al. Oral Chemotherapeutic Agents in Metastatic Hormone-Sensitive Prostate Cancer: A Network Meta-Analysis of Randomized Controlled Trials. *Prostate Int* (2023) 11(3):159-66. Epub 2023/09/25. doi: 10.1016/j.prnil.2023.06.003.

2. Riaz IB, Naqvi SAA, He H, Asghar N, Siddiqi R, Liu H, et al. First-Line Systemic Treatment Options for Metastatic Castration-Sensitive Prostate Cancer: A Living Systematic Review and Network Meta-Analysis. *JAMA Oncol* (2023) 9(5):635-45. Epub 2023/03/03. doi: 10.1001/jamaoncol.2022.7762.

3. Wang L, Li C, Zhao Z, Li X, Tang C, Guan Z, et al. Comparison of Doublet and Triplet Therapies for Metastatic Hormone-Sensitive Prostate Cancer: A Systematic Review and Network Meta-Analysis. *Front Oncol* (2023) 13:1104242. Epub 2023/03/25. doi: 10.3389/fonc.2023.1104242.

4. Wang SS, Bian XJ, Wu JL, Wang BH, Zhang S, Ye DW. Network Meta-Analysis of Combination Strategies in Metastatic Hormone-Sensitive Prostate Cancer. *Asian J Androl* (2024) 26(4):402-8. Epub 2024/04/16. doi: 10.4103/aja20242.

5. Jian T, Zhan Y, Hu K, He L, Chen S, Hu R, et al. Systemic Triplet Therapy for Metastatic Hormone-Sensitive Prostate Cancer: A Systematic Review and Network Meta-Analysis. *Front Pharmacol* (2022) 13:955925. Epub 2022/10/25. doi: 10.3389/fphar.2022.955925.

6. Hoeh B, Garcia CC, Wenzel M, Tian Z, Tilki D, Steuber T, et al. Triplet or Doublet Therapy in Metastatic Hormone-Sensitive Prostate Cancer: Updated Network Meta-Analysis Stratified by Disease Volume. *Eur Urol Focus* (2023) 9(5):838-42. Epub 2023/04/14. doi: 10.1016/j.euf.2023.03.024.

7. Mandel P, Hoeh B, Wenzel M, Preisser F, Tian Z, Tilki D, et al. Triplet or Doublet Therapy in Metastatic Hormone-Sensitive Prostate Cancer Patients: A Systematic Review and Network Meta-Analysis. *Eur Urol Focus* (2023) 9(1):96-105. Epub 20220901. doi: 10.1016/j.euf.2022.08.007.

8. Dou M, Liang H, Liu Y, Zhang Q, Li R, Chen S, et al. Based on Arasens Trial: Efficacy and Safety of Darolutamide as an Emerging Option of Endocrinotherapy for Metastatic Hormone-Sensitive Prostate Cancer—an Updated Systematic Review and Network Meta-Analysis. *J Cancer Res Clin Oncol* (2023) 149(10):7017-27. doi: 10.1007/s00432-023-04658-6.

9. Menges D, Yebyo HG, Sivec-Muniz S, Haile SR, Barbier MC, Tomonaga Y, et al. Treatments for Metastatic Hormone-Sensitive Prostate Cancer: Systematic Review, Network Meta-Analysis, and Benefit-Harm Assessment. *Eur Urol Oncol* (2022) 5(6):605-16. doi: 10.1016/j.euo.2022.04.007.
